# Fish and Meat Intake, Serum Eicosapentaenoic Acid and Docosahexaenoic Acid Levels, and Mortality in Community-Dwelling Japanese Older Persons

**DOI:** 10.3390/ijerph16101806

**Published:** 2019-05-21

**Authors:** Rei Otsuka, Chikako Tange, Yukiko Nishita, Makiko Tomida, Yuki Kato, Tomoko Imai, Fujiko Ando, Hiroshi Shimokata

**Affiliations:** 1 Section of NILS-LSA (National Institute for Longevity Sciences-Longitudinal Study of Aging), National Center for Geriatrics and Gerontology, Aichi 474-8511, Japan; tange@ncgg.go.jp (C.T.); tomida@ncgg.go.jp (M.T.); kyuki@asu.aasa.ac.jp (Y.K.); toimai@dwc.doshisha.ac.jp (T.I.); fujikoa@asu.aasa.ac.jp (F.A.); simokata@nuas.ac.jp (H.S.); 2 Department of Epidemiology of Aging, National Center for Geriatrics and Gerontology, Aichi 474-8511, Japan; nishita@ncgg.go.jp; 3 Faculty of Health and Medical Sciences, Aichi Shukutoku University, Aichi 480-1197, Japan; 4 Faculty of Human Life and Science, Doshisha Women’s College of Liberal Arts, Kyoto 602-0893, Japan; 5 Graduate School of Nutritional Sciences, Nagoya University of Arts and Sciences, Aichi 470-0196, Japan

**Keywords:** fish, meat, DHA, EPA, serum, Japanese, mortality

## Abstract

The associations between meat/fish consumption, docosahexaenoic acid (DHA)/eicosapentaenoic acid (EPA) intakes, and blood DHA/EPA levels, and mortality in Japan were examined as part of the National Institute for Longevity Sciences-Longitudinal Study of Aging: 520 men and 534 women (60–79 years at baseline) were followed from 1997–2017. Nutritional intakes were assessed using a 3-day dietary record and fasting venous blood samples were collected. Serum EPA/DHA concentrations, the EPA/arachidonic acid (ARA) ratio, EPA/DHA intakes, and fish/meat intakes were examined in tertiles as indicator variables, and hazard ratios (HR) were calculated to compare the risk of death across tertiles controlling for sex, age, body mass index, smoking status, alcohol drinking, physical activity, education, employment, and history of diseases. During follow-up (mean 11.7 years), 422 subjects (40.4%) died. The multivariate-adjusted HR for all-cause mortality in subjects in the highest tertile of serum DHA and EPA/ARA ratio was 0.73 (95% confidence intervals (CI): 0.53–0.99) and 0.71 (95% CI: 0.53–0.96) compared with subjects in the lowest tertile, respectively (trend *p* < 0.05). There were no significant associations between mortality and serum EPA/ARA and DHA/EPA intakes. An increased serum DHA level or EPA/ARA ratio might be recommended for longevity to Japanese community dwellers.

## 1. Introduction

Japanese individuals have a long history of eating seafood rich in n-3 polyunsaturated fatty acids (PUFAs), although nutritional intake has changed dramatically over time [1] and become more westernized [2]. Fish consumption has decreased and meat consumption has increased markedly among Japanese individuals in the last 50 to 60 years [1].

Fish consumption, particularly fatty fish, and intake of marine n-3 PUFAs (docosahexaenoic acid (DHA) and eicosapentaenoic acid (EPA)) are thought to play a protective role against coronary heart disease (CHD) [3] and its associated mortality [4]. However, the effects of fish and n-3 PUFA consumption on mortality remain controversial [5,6,7,8]. One possible reason for inconsistent study results is the limited ability of dietary assessments to quantify blood levels of fatty acids. However, blood fatty acid biomarkers can be measured to indicate differences in delayed responses to short- and long-term dietary intakes [9,10]. A study using n-3 series PUFAs in the blood showed that higher n-3 PUFA phospholipid levels were associated with lower all-cause mortality, especially CHD deaths, in older Americans [11]. A low serum DHA, not EPA, level was associated with mortality in Japanese patients with acute decompensated heart failure [12]. However, there is little evidence concerning n-3 series PUFAs and mortality in the general Japanese population.

Mean DHA and EPA intakes and blood DHA/EPA levels are substantially higher in Japanese subjects than in Western subjects [13,14,15,16]. However, the association between blood DHA/EPA levels and mortality in Japanese subjects is unclear.

This study examined the associations of meat/fish consumption, fatty acid intakes including DHA and EPA, and blood DHA/EPA levels, concurrently, with all-cause mortality in community-dwelling Japanese older persons.

## 2. Materials and Methods

### 2.1. Participants in the Baseline Survey

Data for this survey were collected as part of the National Institute for Longevity Sciences-Longitudinal Study of Aging (NILS-LSA). In this project, the normal aging process was assessed over time using detailed questionnaires and medical checkups, anthropometric measurements, physical fitness tests, and nutritional examinations. Participants in the NILS-LSA included randomly selected age- and sex-stratified individuals from a pool of non-institutionalized residents in the NCGG neighborhood areas of Obu City and Higashiura Town in the Aichi Prefecture. The first wave of the NILS-LSA was conducted from November 1997 to April 2000 and comprised of 2267 participants (1139 men, 1128 women; age range, 40–79 years). They were followed up biennially from the second study-wave (2000–2002) to the seventh study-wave (July 2010–July 2012). Details of the NILS-LSA study have been reported elsewhere [17]. The study protocol was approved by the Committee of Ethics of Human Research of the National Center for Geriatrics and Gerontology (No. 899-3). Written, informed consent was obtained from all subjects.

### 2.2. Follow-Up and Study Subjects

The residence records of all participants were reviewed to determine their vital status and information was obtained from the local government regarding which participants moved away from their local residence to other areas. To clarify the cause of death, the National Vital Statistics records that were available until the end of December 2017 were used. The underlying cause of death was coded according to the tenth International Classification of Diseases in 2013. Deaths were confirmed by computer matching of data from the National Vital Statistics records using neighborhood areas, sex, date of birth, and death as key codes.

Of the 2267 participants, participants aged 60 years and older at baseline (*n* = 1133) were selected. The following were excluded: (1) Those who fasted <12 h or were unable to supply a sufficient volume of blood (*n* = 20); (2) those with an energy intake less than 500 kcal/day as measured by dietary records, or no dietary records (*n* = 42); and (3) those who had missing data for controlled variables (*n* = 18). A total of 520 men and 534 women between 60 and 79 years of age at baseline were available for analysis.

### 2.3. Blood Sampling and Serum Fatty Acid Analysis

On enrollment in the baseline study, venous blood was collected early in the morning after fasting for at least 12 h. Blood samples were centrifuged at 3500 × *g* for 15 min. The serum was refrigerated before analysis for fatty acid content. Serum DHA, EPA, and arachidonic acid (ARA) concentrations were measured by gas-liquid chromatography at a clinical laboratory (SRL, Tokyo, Japan). Briefly, total lipids in the serum were extracted using the Folch procedure, and fatty acids were then methylated with BF3/methanol. Transesterified fatty acids were then analyzed using a gas chromatograph (GC-17A; Shimadzu, Kyoto, Japan) with a capillary column (Omegawax 250; Supelco, Bellefonte, PA, USA). The weights of DHA and EPA (g/mL) as fatty acid concentrations were identified by comparison with known standards. Intra- and inter-assay precision and accuracy values (coefficient of variation [CV]) were 3.2 and 5.8 CV% for ARA, 2.7 and 6.9 CV% for EPA, and 1.9 and 6.9 CV% for DHA, respectively [18]. In the statistical analyses, serum EPA, DHA, ARA concentrations, and the EPA/ARA ratio, which predict future cardiovascular events [19], were selected as serum fatty acid factors.

### 2.4. Nutritional Assessments

Nutritional intakes were assessed using a 3-day dietary record without any supplements after participation in the baseline study. The dietary record was completed over 3 continuous days (2 weekdays and 1 weekend day) [19], and most subjects completed it at home and returned records within 1 month. Food was weighed separately on a scale (1-kg kitchen scales; Sekisui Jushi, Tokyo, Japan) before being cooked, or portion sizes were estimated. Subjects used a disposable camera (27 shots; Fuji Film, Tokyo, Japan) to take photos of meals before and after eating. Dietitians used these photos to complete missing data and telephoned subjects to resolve any discrepancies or obtain further information when necessary. Averages for 3-day food and nutrient intakes (including alcohol intake) were calculated according to the Standard Tables of Foods Composition in Japan 2010 and other sources [20,21].

### 2.5. Other Measurements

History of diseases, education (≤9, 10–12, or ≥13 years of school), employment (unemployed, regular employment, or non-regular employment), and smoking status (yes/no) were collected using self-reported questionnaires, and medical doctors or trained staff confirmed the information. Concerning diseases, information about past and current cancer, heart disease, stroke, hypertension, dyslipidemia, and diabetes mellitus was collected in 4 categories: (1) None; (2) on medication; (3) previously medicated; and (4) not treated. These illnesses were then re-categorized into 2 categories (yes or no; yes included (2) on medication, (3) previously medicated, and (4) not treated, and no included (1) none).

Body mass index (BMI) was calculated as weight in kilograms divided by the square of the height in meters. Physical activity was assessed by the metabolic equivalents (METs) score (a multiple of the resting metabolic rate), with participants interviewed by trained interviewers using a semi-quantitative assessment method to assess participants’ levels of habitual physical activity during leisure time, on the job, and sleeping hours [22]. These measurements were assessed in the baseline survey.

### 2.6. Statistical Analysis

Fish and meat intakes, fatty acid intakes including EPA, DHA, and ARA, and serum EPA, DHA, ARA concentrations, and the EPA/ARA ratio were examined in sex-stratified tertiles as indicator variables. Men generally consume more food than women, and the ratio of men would be higher in higher intake groups (i.e., higher tertiles) if tertiles were calculated with men and women together; therefore, sex-stratified tertiles were calculated for each explanatory variable.

The relationships of fish and meat intakes, EPA and DHA intakes, serum EPA, DHA, and ARA concentrations, and the EPA/ARA ratio with the survival rate (all-cause mortality) were assessed using Kaplan–Meier survival curves (Supplementary Figures S1 and S2). The hazard ratios (HR) and 95% confidence intervals (CI) for outcomes were calculated by tertiles using a Cox proportional hazards model. Tests for trends were based on modeling in each tertile category. Follow-up time (years) was calculated by the length of time (days) that had elapsed since the day each subject participated in the baseline study. The end point of the study was calculated as the time at death or the latest date of the adjudicated follow-up. The confounding variables were sex, age, BMI, smoking status, alcohol drinking, physical activity, education, employment, and history of cancer, heart disease, stroke, hypertension, dyslipidemia, and diabetes mellitus.

Based on the main results (i.e., the significant association between serum DHA and mortality), differences in proportions according to tertiles of serum DHA concentrations were assessed using the χ^2^ test. Comparisons between continuous variables according to tertiles of serum DHA concentrations were performed by one-way analysis of variance. The Tukey test was used to adjust for multiple comparisons.

All reported *p* values are two-sided, and a *p* value less than 0.05 was considered significant. All statistical analyses were conducted using Statistical Analysis System software version 9.3 (SAS Institute, Cary, NC, USA).

## 3. Results

During the follow-up from the baseline survey to December 2017, 422 subjects (40.4%) died; their mean (standard deviation (SD)) follow-up was 11.7 (4.8) years, whereas that of the survivors was 17.8 (3.2) years. Of the 422 deaths, 31.3% (*n* = 132) were caused by cancer, 14.2% (*n* = 60) by heart disease, and 11.4% (*n* = 47) by stroke.

Table 1 shows the baseline characteristics of the participants. More than 37% of subjects had a history of hypertension and more than 21% of subjects had a history of dyslipidemia. Mean (SD) daily fish, meat, EPA, and DHA intakes were 98.8 (50.5) g, 51.0 (31.7) g, 319.1 (240.3) mg, and 569.4 (382.5) mg, respectively.

Table 2 shows the multivariate-adjusted HR for all-cause mortality of fish, meat, and fatty acid intakes. Although there were no significant associations, the HR for mortality of the middle and highest tertiles of fatty acid intake were 0.80 (95% CI: 0.59–1.08) and 0.74 (95% CI: 0.55–1.01), respectively, compared with the lowest tertile of fatty acid intake. The HR for mortality of the highest tertile of saturated fatty acid intake was 0.73 (95% CI: 0.53–0.99) compared with the lowest tertile of saturated fatty acid intake (trend *p* < 0.05). Fish, meat, and unsaturated fatty acid intakes, including EPA and DHA intakes, were not associated with mortality.

Multivariate-adjusted HR for all-cause mortality according to serum EPA, DHA, ARA, and the EPA/ARA ratio are shown in Table 3. Although there were no significant associations between mortality and serum EPA and ARA, the HR for mortality of the highest tertile of serum DHA was 0.73 (95% CI: 0.53–0.99) compared with the lowest tertile of serum DHA. In addition, the HR for mortality of the highest tertile of the serum EPA/ARA ratio was 0.71 (95% CI: 0.53–0.96) compared with the lowest tertile of the serum EPA/ARA ratio.

Table 4 shows the baseline characteristics of the participants by tertiles of serum DHA. Subjects with higher serum DHA levels were more likely to have a higher BMI and to consume more fish, DHA, EPA, and ARA in their diet. On the other hand, those with higher serum DHA levels were less likely to engage in physical activity.

## 4. Discussion

These results suggest the beneficial effect of higher serum DHA levels on all-cause mortality in Japanese older persons whose DHA and EPA intake/serum DHA and EPA levels are higher than those of Western people. On the other hand, fish, meat, EPA, and DHA intakes were not associated with mortality.

Many previous studies have focused on fish and meat consumption and health outcomes such as breast cancer [23], pancreatic cancer [24], and cerebrovascular disease mortality [25]. One study suggested that both meat and fish intake reduced the incidence of cerebrovascular disease in Japanese persons [25]. On the other hand, a meta-analysis indicated that consumption of red and/or processed meat increased the risk of stroke [26], and consumption of processed meat increased the risk of CHD [27]. Meat is a major source of protein for humans, but saturated fatty acids contained in raw meat or sodium in processed meat are thought to have a negative effect on coronary artery disease [28]. In the present study, fatty acid intake, especially saturated fatty acid intake, was negatively associated with mortality, but meat was not associated with mortality.

Meta-analyses of 12 prospective cohort studies reported that the saturated fat intake was not associated with all-cause mortality [29]. Another prospective cohort study conducted in 18 countries reported that total fat and each type of fat, including saturated fatty acid, intake were associated with lower all-cause mortality [30]. They suggested that the reasons for the negative association between fat intake and mortality depended on the study population. In that study [30], more than half of the participants consumed a high carbohydrate diet (at least 60% of energy), the subjects lived mainly in Asia, and the percentage was higher than in most previous studies done in North America and Europe. In the present study, Japanese participants also consumed a high carbohydrate diet (mean: 58.7% of energy, data not shown), therefore, the results of the negative association between saturated fatty acid intake and mortality are consistent in Asian people. In other words, the results indicated that higher saturated fatty acid intake in Asian people was not necessarily associated with a higher risk of mortality.

In the present study, higher serum DHA levels (*p* trend = 0.047) and a higher serum EPA/ARA ratio (*p* trend = 0.02) were negatively associated with all-cause mortality, though higher serum EPA levels were not significantly negatively associated with mortality. DHA and EPA are thought to be important for the prevention of cardiovascular disease, and DHA has effects on membrane properties and cell signaling [31], whereas EPA has a more pronounced effect on eicosanoid production [32]. In a previous study, low serum DHA, not EPA, was associated with mortality in Japanese patients with acute decompensated heart failure [12]. In addition, a meta-analysis of general populations including the Japanese reported that n-3 polyunsaturated fatty acid intake as well as blood DHA and EPA were negatively associated with mortality [33]. Among Japanese patients undergoing percutaneous coronary intervention, the higher serum EPA/ARA ratio group (>0.40) had a significantly lower incidence of major cardiac events than the lower EPA/ARA ratio group (≤0.40) [19]. In the present study, the median values of the first (lower) and second (middle) tertiles in the serum EPA/ARA ratio group were 0.3 and 0.4, respectively. The precise mechanism was unknown, but the blood level of DHA and the EPA/ARA ratio might be protective factors for longevity through membrane properties, cell signaling, and lower cardiac events.

There were no positive associations between DHA and EPA intakes and the risk of mortality. The lack of findings might be caused by difficulties with dietary assessments. Japanese people usually eat one or two fish dishes per day, and the median intake/day of subjects in the present study was 90 g for fish and 48 g for meat. Even if the Japanese consume fish daily, within individual variations of n-3 PUFAs such as EPA or DHA that were high, and the accuracy of n-3 PUFA values assessed by 3-day dietary records was estimated to have a 28% coefficient of variation among Japanese people [34]. The limited ability of dietary assessments to quantify blood levels of fatty acids is a major possible reason for previous inconsistent results regarding n-3 PUFA consumption and risk of mortality [5,6,7,8]. Another possible reason could be confounding factors such as high blood pressure or cardiovascular disease. In the present study, adjustments were made for many variables, including cancer, heart disease, stroke, hypertension, dyslipidemia, and diabetes mellitus, and that might have led to over-adjustment. More precise and careful control of confounders may be necessary.

Several limitations to the present study warrant consideration. Dietary factors and serum fatty acid levels were assessed from one nutritional assessment and a single blood sampling at baseline. Food intake is easily changeable and affected by various factors with aging [35,36], though these variations could not be considered during the follow-up period. In addition, the number of cause-specific deaths was not large, and no significant associations were found between the serum DHA level and cause-specific disease mortality (data not shown). Larger prospective studies are needed to examine these fatty acids and cause-specific disease mortality. In addition, attrition bias may have attenuated the results. Furthermore, since DHA and EPA intakes/serum levels are substantially higher in Japanese than in Western subjects [13], prudent interpretation of the results is needed (i.e., low DHA/EPA levels among Japanese subjects might not mean low levels among Western subjects). Thus, the results of this study cannot necessarily be applied to Western populations.

In previous reports, we showed the positive correlations between serum DHA composition and DHA intake in the same cohort study [37]. In the present study, although there was no negative association between fish/DHA intake and mortality, intake of more fish/DHA would increase serum DHA levels.

## 5. Conclusions

The findings of this study indicate that an increase in serum DHA levels or the EPA/ARA ratio might be recommended for longevity to Japanese community dwellers.

## Figures and Tables

**Table 1:** Baseline characteristics of participants (*n* = 1054).

Characteristics	Range			
	Min	Max	Mean	SD
Men (*n*, %)	-		520, 49.3	
Age (years)	60	79	68.6	5.5
Body mass index (kg/m^2^)	14.1	42.4	22.9	3.1
Alcohol (mL/day)	0	116.7	7.5	13.9
Physical activity (1000 METs × h/day)	26.6	62.8	34.5	3.8
Current smoker (*n*, %)	-		186, 17.7	
Education				
≤ 9 years (*n*, %)	-		507, 48.1	
10–12 years (*n*, %)	-		379, 36.0	
≥ 13 years (*n*, %)	-		168, 15.9	
Employment				
Unemployed (*n*, %)	-		733, 69.5	
Regular employment (*n*, %)	-		201, 19.1	
Non-regular employment (*n*, %)	-		120, 11.4	
Diseases				
Cancer (*n*, %)	-		58, 5.5	
Heart disease (*n*, %)	-		181, 17.2	
Stroke (*n*, %)	-		49, 7.1	
Hypertension (*n*, %)	-		394, 37.4	
Dyslipidemia (*n*, %)	-		228, 21.6	
Diabetes (*n*, %)	-		119, 11.3	
Dietary intake				
Fish intake (g/day)	0.0	346.2	98.8	50.5
Meat intake (g/day)	0.0	222.7	51.0	31.7
EPA intake (mg/day)	6.6	1638.8	319.1	240.3
DHA intake (mg/day)	23.0	2722.5	569.4	382.5
ARA intake (mg/day)	18.9	541.5	167.4	64.9
Serum fatty acid				
EPA (μg/mL)	5.8	269.1	70.7	37.0
DHA (μg/mL)	30.2	460.6	152.5	47.1
ARA (μg/mL)	61.8	337.4	149.2	35.9
EPA/ARA ratio	0.1	2.3	0.5	0.3

Min, minimum; Max, maximum; SD, standard deviation; METs, metabolic equivalents; DHA, docosahexaenoic acid; EPA, eicosapentaenoic acid; ARA, arachidonic acid.

**Table 2:** Multivariate-adjusted hazard ratios ^1^ for all-cause mortality of fish, meat, and fatty acid intakes (*n* = 1054).

Explanatory Variable	Tertile 1				Tertile 2				Tertile 3				*p* trend
	Median		Cases *n* /Alive *n*	HR (ref)	Median		Cases *n* /Alive *n*	HR (95% CI)	Median		Cases n /Alive n	HR (95% CI)	
Fish intake	55.0	g/day	142/208	1	89.5	g/day	142/208	1.16 (0.85–1.58)	141.4	g/day	138/216	1.20 (0.89–1.63)	0.23
Meat intake	20.0	g/day	159/188	1	46.7	g/day	136/215	0.77 (0.57–1.03)	80.0	g/day	127/229	0.81 (0.60–1.11)	0.19
Fatty acid intake	31.9	g/day	171/179	1	43.5	g/day	134/217	0.80 (0.59–1.08)	57.2	g/day	117/236	0.74 (0.55–1.01)	0.06
Saturated fatty acid intake	9.7	g/day	160/190	1	14.0	g/day	143/208	0.75 (0.55–1.01)	19.5	g/day	119/234	0.73 (0.53–0.99)	0.04
Unsaturated fatty acid intake	21.2	g/day	171/179	1	29.2	g/day	134/217	0.72 (0.53–0.97)	38.9	g/day	117/236	0.78 (0.57–1.05)	0.10
n-6 polyunsaturated fatty acid intake	6.8	g/day	160/190	1	9.6	g/day	140/211	0.69 (0.50–0.93)	12.6	g/day	122/231	0.80 (0.59–1.07)	0.13
ARA intake	104.4	mg/day	159/191	1	159.0	mg/day	134/217	0.98 (0.73–1.33)	226.1	mg/day	129/224	0.95 (0.69–1.30)	0.74
n-3 polyunsaturated fatty acid intake	1.5	g/day	166/184	1	2.2	g/day	122/229	0.77 (0.57–1.05)	3.3	g/day	134/219	0.83 (0.61–1.12)	0.22
EPA intake	101.7	mg/day	162/188	1	268.8	mg/day	120/231	0.85 (0.63–1.16)	530.3	mg/day	140/213	0.96 (0.71–1.30)	0.78
DHA intake	226.1	mg/day	160/190	1	499.4	mg/day	130/221	0.85 (0.62–1.16)	864.2	mg/day	132/221	0.95 (0.70–1.28)	0.72

^1^ HR adjusted for sex, baseline age, body mass index, smoking status, alcohol drinking, physical activity, education, employment, and history of cancer, heart disease, stroke, hypertension, dyslipidemia, and diabetes mellitus. HR, hazard ratios; CI, confidence intervals; EPA, eicosapentaenoic acid; DHA, docosahexaenoic acid; ARA, arachidonic acid.

**Table 3:** Multivariate-adjusted hazard ratios ^1^ for all-cause mortality of serum EPA, DHA, and ARA (*n* = 1054).

Explanatory Variable	Tertile 1				Tertile 2				Tertile 3				*p* trend
	Median		Cases n /Alive *n*	HR (ref)	Median		Cases *n* /Alive *n*	HR (95% CI)	Median		Cases *n* /Alive *n*	HR (95% CI)	
Serum EPA	38.1	μg/mL	150/200	1	62.9	μg/mL	133/218	0.82 (0.61–1.10)	101.9	μg/mL	139/214	0.81 (0.60–1.09)	0.17
Serum DHA	111.6	μg/mL	151/197	1	147.1	μg/mL	143/210	0.82 (0.61–1.10)	191.0	μg/mL	128/225	0.73 (0.53–0.99)	0.047
Serum ARA	116.7	μg/mL	136/213	1	144.0	μg/mL	156/195	1.47 (1.10–1.98)	184.2	μg/mL	130/224	0.90 (0.64–1.24)	0.51
Serum EPA/ARA	0.3		141/212	1	0.4		128/223	0.62 (0.45–0.84)	0.7		141/212	0.71 (0.53–0.96)	0.02

^1^ HR adjusted for sex, baseline age, body mass index, smoking status, alcohol drinking, physical activity, education, employment, and history of cancer, heart disease, stroke, hypertension, dyslipidemia, and diabetes mellitus. HR, hazard ratios; CI, confidence intervals; EPA, eicosapentaenoic acid; DHA, docosahexaenoic acid; ARA, arachidonic acid.

**Table 4:** Baseline characteristics of participants by tertiles of serum DHA (*n* = 1054).

Characteristics	Tertile 1		Tertile 2		Tertile 3		*p* ^1^	*p* ^2^
	Mean	SD	Mean	SD	Mean	SD		
Number of subjects	348		353		353			
Serum DHA (μg/mL)	108.0	17.4	146.5	10.4	202.3	41.8	<0.0001	*, **, ***
Men (*n*, %)	172, 49.3		174, 49.3		174, 49.3		0.99	
Age (years)	68.9	5.4	68.6	5.6	68.4	5.4	0.47	
Body mass index (kg/m^2^)	22.7	3.1	22.6	3.4	23.3	2.9	0.01	*, **
Alcohol (mL/day)	6.3	12.9	7.5	13.8	8.7	14.8	0.08	
Physical activity (1000 METs × h/day)	34.8	3.9	34.6	4.0	34.0	3.3	0.01	**
Current smoker (n, %)	59, 17.0		70, 19.8		57, 16.2		0.40	
Education								
≤ 9 years (*n*, %)	175, 50.3		171, 49.3		158, 44.8		0.48	
10–12 years (*n*, %)	121, 34.8		119, 33.7		139, 39.4			
≥ 13 years (*n*, %)	52, 14.9		60, 17.0		56, 15.9			
Employment								
Unemployed (*n*, %)	240, 69.0		245, 69.4		248, 70.3		0.94	
Regular employment (*n*, %)	69, 19.8		63, 17.9		69, 19.6			
Non-regular employment (*n*, %)	39, 11.2		45, 12.8		36, 10.2			
Diseases								
Cancer (*n*, %)	23, 6.6		19, 5.4		16, 4.6		0.49	
Heart disease (*n*, %)	64, 18.4		62, 17.6		55, 15.6		0.60	
Stroke (*n*, %)	16, 6.9		12, 5.0		21, 9.4		0.19	
Hypertension (*n*, %)	128, 36.8		123, 34.8		143, 40.5		0.29	
Dyslipidemia (*n*, %)	54, 15.5		67, 19.0		107, 30.3		<0.0001	
Diabetes (*n*, %)	41, 11.8		35, 9.9		43, 12.2		0.60	
Dietary intake								
Fish intake (g/day)	89.0	46.5	96.3	44.5	110.8	57.1	<0.0001	*, **
Meat intake (g/day)	49.4	29.6	51.3	32.7	52.1	32.7	0.52	
EPA intake (mg/day)	276.6	220.9	304.9	202.7	375.4	280.0	<0.0001	*, **
DHA intake (mg/day)	500.8	338.2	547.8	332.8	658.7	448.3	<0.0001	*, **
ARA intake (mg/day)	158.3	59.2	165.8	60.1	178.1	73.1	<0.001	*, **

^1^ For continuous variables, one-way analysis of variance was used; for categorical variables, the χ^2^ test was used. ^2^ For continuous variables, the Tukey test was used for multiple comparisons (* Tertile 1 vs. Tertile 2 *p* < 0.05, ** Tertile 1 vs. Tertile 3 *p* < 0.05, *** Tertile 2 vs. Tertile 3 *p* < 0.05). DHA, docosahexaenoic acid; SD, standard deviation; METs, metabolic equivalents; EPA, eicosapentaenoic acid; ARA, arachidonic acid.

## Supplementary Materials

The following are available online at https://www.mdpi.com/1660-4601/16/10/1806/s1, Figure S1: Survival curves according to tertiles of fish, meat, DHA, and EPA intake, Figure S2: Survival curves according to tertiles of serum DHA, EPA, ARA, and the EPA/ARA ratio.
