# Supplementary material for: Fish and Meat Intake, Serum Eicosapentaenoic Acid and Docosahexaenoic Acid Levels, and Mortality in Community-Dwelling Japanese Older Persons
Source: Int J Environ Res Public Health. 2019 May 21;16(10):1806. doi: 10.3390/ijerph16101806 (PMC6572518; doi:10.3390/ijerph16101806)
Supplement: Supplementary file 1 [file ijerph-16-01806-s001.pdf]

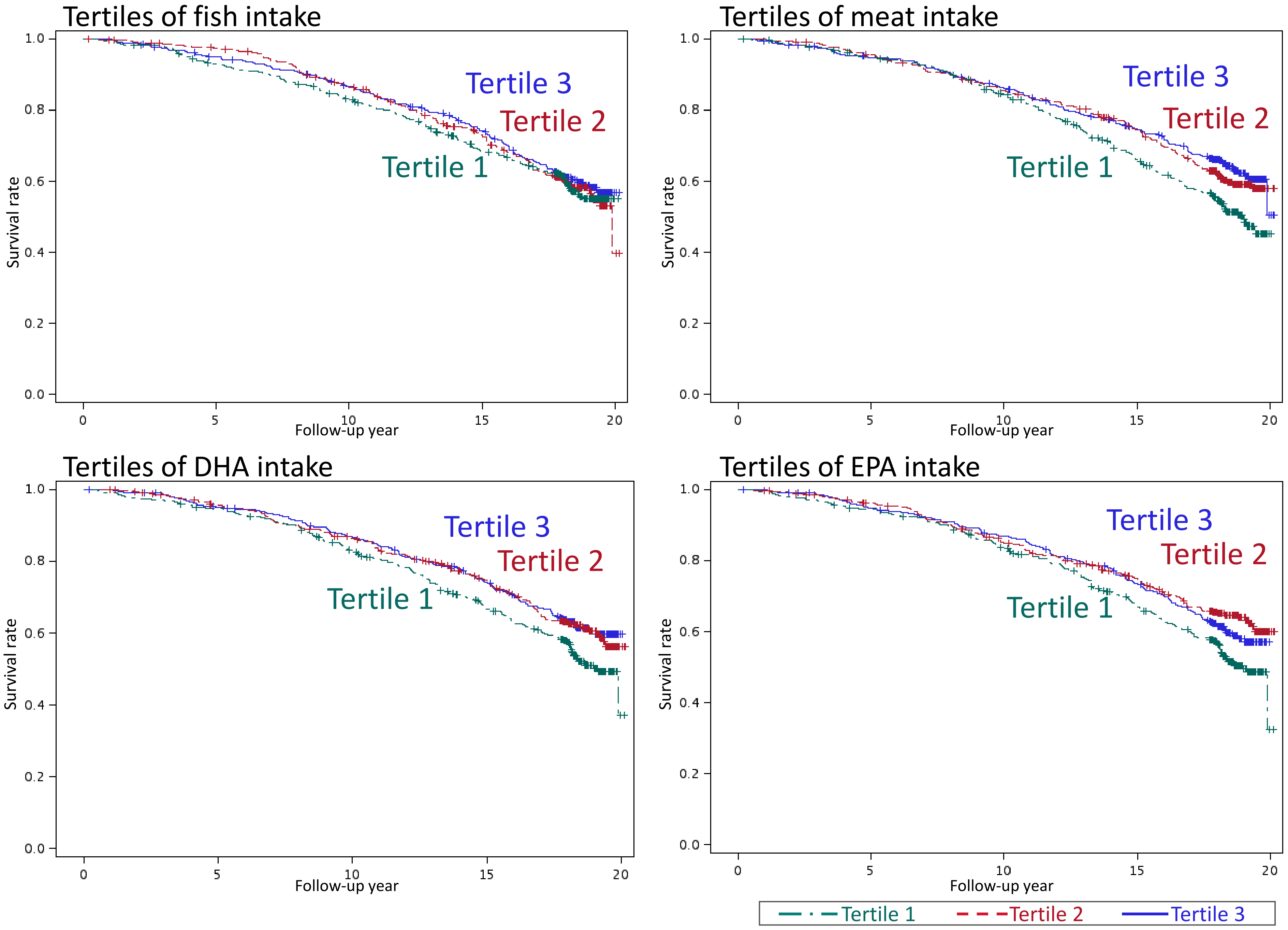

Supplementary Figure S1. Survival curves according to tertiles of fish, meat, DHA, and EPA intake

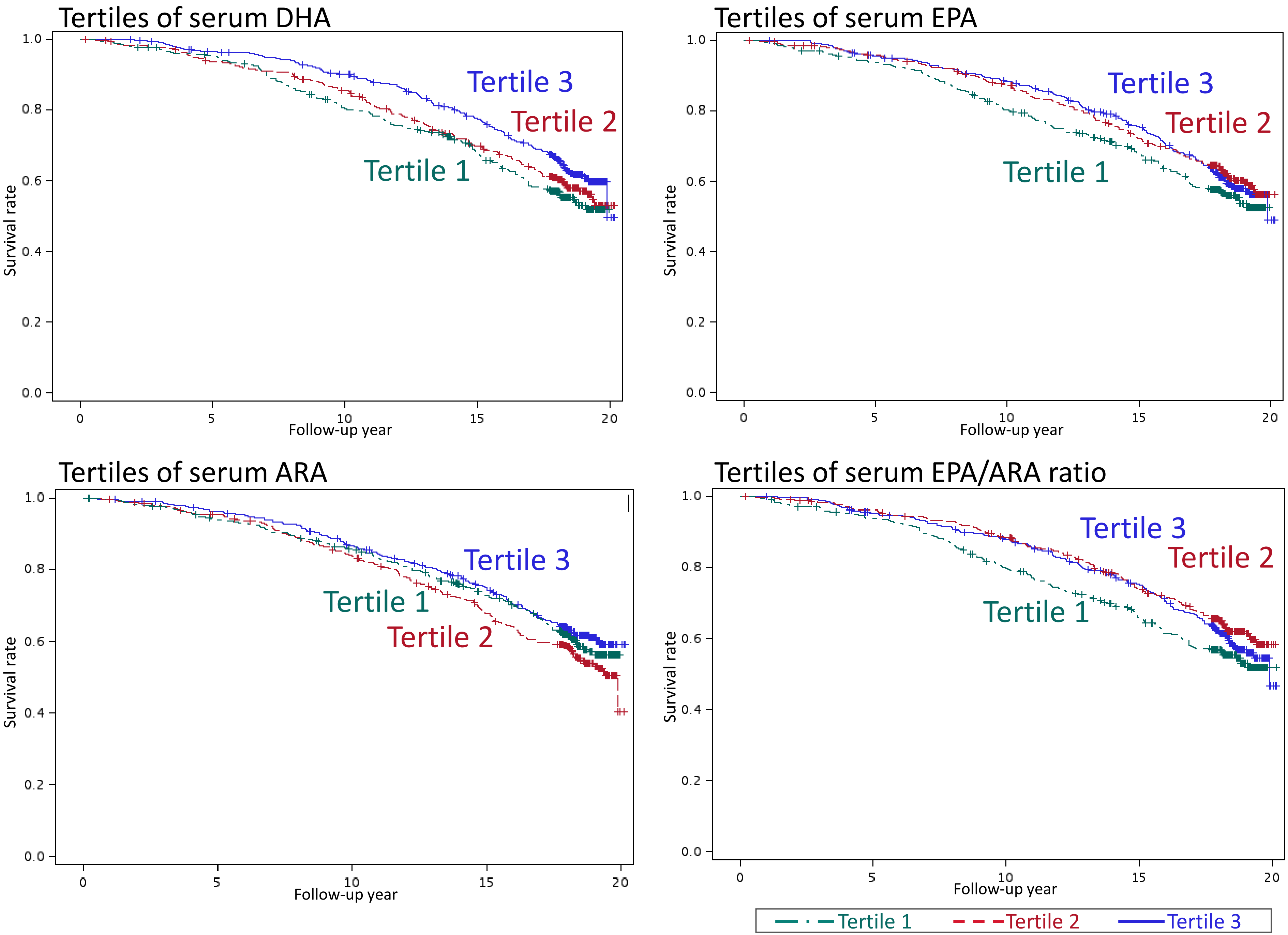

Supplementary Figure S2. Survival curves according to tertiles of serum DHA, EPA, ARA, and the EPA/ARA ratio
